# Machine learning model to predict hypotension after starting continuous renal replacement therapy

**DOI:** 10.1038/s41598-021-96727-4

**Published:** 2021-08-25

**Authors:** Min Woo Kang, Seonmi Kim, Yong Chul Kim, Dong Ki Kim, Kook-Hwan Oh, Kwon Wook Joo, Yon Su Kim, Seung Seok Han

**Affiliations:** Department of Internal Medicine, Seoul National University College of Medicine, 103 Daehak-ro, Jongno-gu, Seoul, 03080 Korea

**Keywords:** Kidney, Kidney diseases, Renal replacement therapy

## Abstract

Hypotension after starting continuous renal replacement therapy (CRRT) is associated with worse outcomes compared with normotension, but it is difficult to predict because several factors have interactive and complex effects on the risk. The present study applied machine learning algorithms to develop models to predict hypotension after initiating CRRT. Among 2349 adult patients who started CRRT due to acute kidney injury, 70% and 30% were randomly assigned into the training and testing sets, respectively. Hypotension was defined as a reduction in mean arterial pressure (MAP) ≥ 20 mmHg from the initial value within 6 h. The area under the receiver operating characteristic curves (AUROCs) in machine learning models, such as support vector machine (SVM), deep neural network (DNN), light gradient boosting machine (LGBM), and extreme gradient boosting machine (XGB) were compared with those in disease-severity scores such as the Sequential Organ Failure Assessment and Acute Physiology and Chronic Health Evaluation II. The XGB model showed the highest AUROC (0.828 [0.796–0.861]), and the DNN and LGBM models followed with AUROCs of 0.822 (0.789–0.856) and 0.813 (0.780–0.847), respectively; all machine learning AUROC values were higher than those obtained from disease-severity scores (AUROCs < 0.6). Although other definitions of hypotension were used such as a reduction of MAP ≥ 30 mmHg or a reduction occurring within 1 h, the AUROCs of machine learning models were higher than those of disease-severity scores. Machine learning models successfully predict hypotension after starting CRRT and can serve as the basis of systems to predict hypotension before starting CRRT.

## Introduction

Continuous renal replacement therapy (CRRT) is an important therapeutic option for severe acute kidney injury with unstable vital signs in critically ill patients. Their outcomes are much worse because they frequently have several comorbidities and imbalanced fluid and electrolytes^1–4^. Although CRRT is started at the right time, complications such as hemodynamic and metabolic crises can aggravate patient outcomes^5–8^. Accordingly, it should be determined which patient subset will benefit from CRRT without complication.

To accomplish this, early prediction of the CRRT-related complication risk is needed in clinical practice, but it has been inadequately resourced. The precise prediction of complications during CRRT may be difficult because several other conditions have interactive and complex effects on the risk^1,2^. Heterogeneous features of patients may also complicate precise prediction. Artificial intelligence may have a role in this difficult assignment, particularly when the numbers of clinical features and their potential interactions rise^9^. Regarding this issue, we previously used machine learning models to predict the mortality risk in patients starting CRRT and found that the model performance was better than conventional disease-severity scores such as the Sequential Organ Failure Assessment (SOFA), the Acute Physiologic Assessment and Chronic Health Evaluation (APACHE) II, and the abbreviated mortality scoring system for acute kidney injury with CRRT (MOSAIC)^10^. The study results may widen the area of machine learning applicability, particularly in the field of critical care using CRRT. Nevertheless, there are still a number of issues to be addressed in determining whether machine learning can predict other CRRT-related outcomes better than conventional scoring systems.

Hypotension frequently occurs after starting CRRT in up to 40% of cases^11,12^. This complication may be attributable to disease severity and sometimes to the labored setting of CRRT, and thus, it may not be easily predicted, as described above^13^. Neither models have been developed nor have conventional scoring models been tested to predict hypotension after CRRT. Herein, we addressed whether machine learning models successfully predicted hypotension in a cohort of CRRT in comparison to conventional scoring models.

## Results

### Baseline characteristics

The mean age of all patients was 64 ± 15 years old, and 61.4% were male. Their systolic blood pressure (SBP), diastolic blood pressure (DBP), and mean arterial pressure (MAP) values were 114 ± 28, 59 ± 16, and 77 ± 17 mmHg, respectively. The target dose of CRRT was 40.7 ± 13.1 ml/kg/hr. Information on other features are shown in Table S1. None of the features differed between the training and testing sets.

### Association between hypotension and mortality

The prevalence of hypotension which was defined as a reduction in MAP ≥ 20 mmHg and ≥ 30 mmHg within 6 h were 29% (n = 673) and 14% (n = 335), respectively. When the timeframe was within 1 h, the prevalence of a reduction in MAP ≥ 20 mmHg and ≥ 30 mmHg were 10% (n = 238) and 4% (n = 97), respectively. Figure S1 shows the nonlinear relationship between the odds ratio for ICU mortality and the reduction in MAP after CRRT. The patients with a larger decrease in MAP within 6 h or 1 h showed higher risk of intensive care unit (ICU) mortality than their counterparts.

### Performance of machine learning models

**Figure 1:**
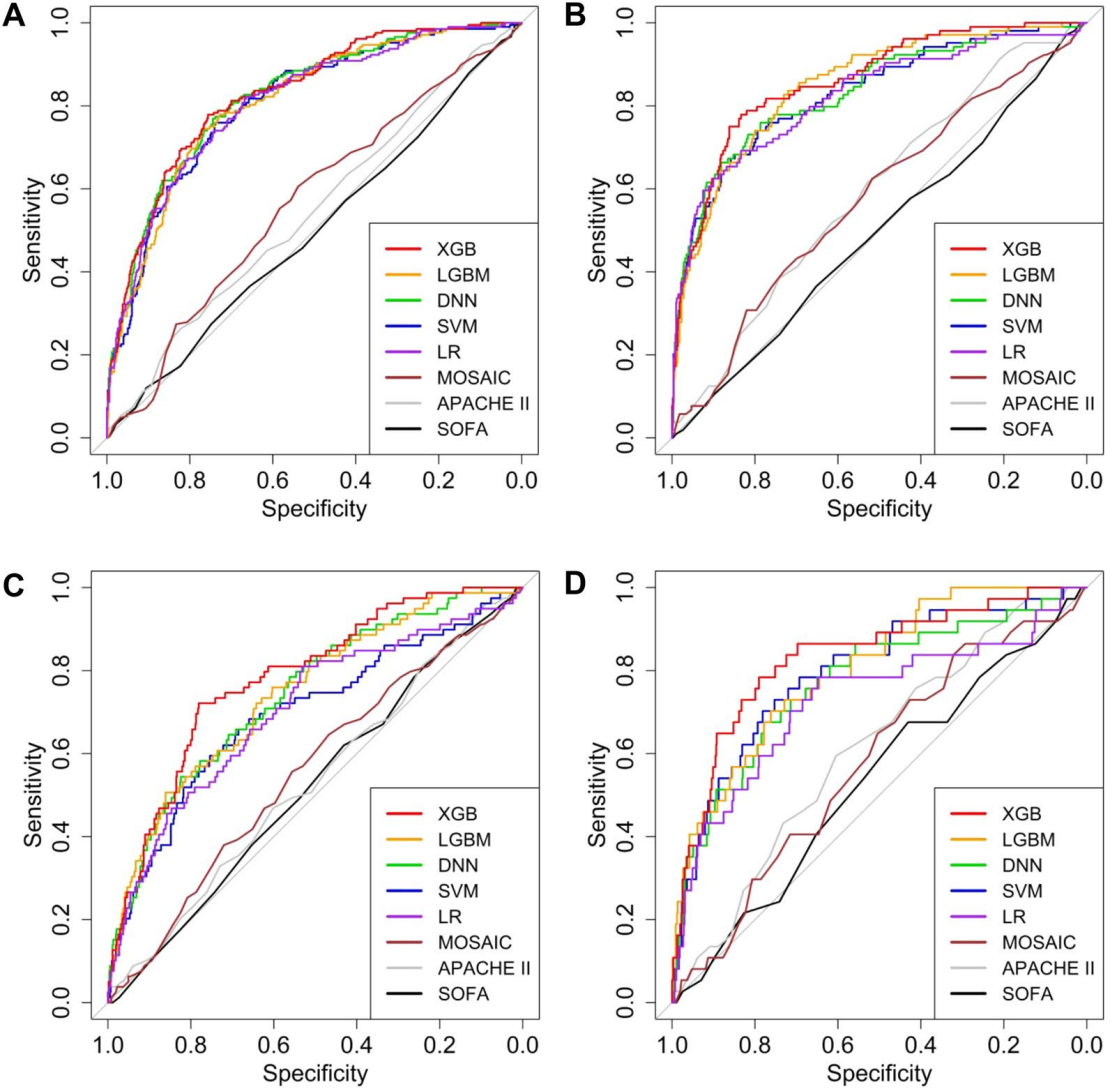
Receiver operating characteristic curves of models in predicting a reduction in mean arterial pressure ≥ 20 mmHg (**A**, **C**) and ≥ 30 mmHg (**B**, **D**) within 6 h (**A**, **B**) and 1 h (**C**, **D**).

When the machine learning models for a reduction in MAP ≥ 20 mmHg within 6 h were evaluated by area under the receiver operating characteristic curves (AUROCs), the extreme gradient boosting machine (XGB) model had the highest value of 0.828 (0.796–0.861), and the deep neural network (DNN) model had the second highest with an AUROC of 0.822 (0.789–0.856) (Table 1). All of the AUROC values in machine learning models were higher than those obtained from SOFA, APACHE II, and MOSAIC scores (*P*s < 0.001). When the outcome was defined as a reduction in MAP ≥ 30 mmHg within 6 h, the best model was the XGB with an AUROC of 0.861 (0.822–0.900). The light gradient boosting machine (LGBM) models achieved the next highest AUROC value of 0.845 (0.802–0.888). Even in this outcome, the machine learning models demonstrated superior performance to the SOFA, APACHE II, and MOSAIC scores (*P*s < 0.001). The plots of AUROCs support these results (Fig. 1). When other outcomes were used such as setting the timeframe to within 1 h or nadir MAP of 65 or 55 mmHg, the XGB model had the higher AUROC values than the SOFA, APACHE II, and MOSAIC scores (*P*s < 0.001) (hypotension within 1 h in Table S2; nadir MAP in Table S3).

**Table 1:** Area under the receiver operating characteristic curves of models predicting hypotension within 6 h.

Models	Outcomes									
	MAP Δ20	P*	*P*^†^	*P*^‡^	*P*^§^	MAP Δ30	P*	*P*^†^	*P*^‡^	*P*^§^
SOFA	0.500 (0.453–0.547)					0.496 (0.435–0.557)				
APACHE II	0.546 (0.499–0.593)					0.592 (0.535–0.649)				
MOSAIC	0.568 (0.522–0.615)					0.578 (0.518–0.638)				
LR	0.809 (0.774–0.844)					0.824 (0.775–0.873)				
SVM	0.807 (0.772–0.842)	< 0.001	< 0.001	< 0.001	0.686	0.830 (0.784–0.876)	< 0.001	< 0.001	< 0.001	0.536
DNN	0.822 (0.789–0.856)	< 0.001	< 0.001	< 0.001	0.601	0.835 (0.789–0.881)	< 0.001	< 0.001	< 0.001	0.755
LGBM	0.813 (0.780–0.847)	< 0.001	< 0.001	< 0.001	0.768	0.845 (0.802–0.888)	< 0.001	< 0.001	< 0.001	0.216
XGB	0.828 (0.796–0.861)	< 0.001	< 0.001	< 0.001	0.440	0.861 (0.822–0.900)	< 0.001	< 0.001	< 0.001	0.253

*Compared with the APACHE II model.

^†^Compared with the SOFA model.

^‡^Compared with the MOSAIC model.

^§^Compared with the LR model.

*MAP* mean arterial pressure, *MAP Δ20* reduction in MAP ≥ 20 mmHg from the initial value, *MAP Δ30* reduction in MAP ≥ 30 mmHg from the initial value, *SOFA* Sequential Organ Failure Assessment, *APACHE* Acute Physiology and Chronic Health Evaluation, *MOSAC* Mortality Scoring system for AKI with CRRT, *LR* Logistic regression, *SVM* support vector machine, *DNN* deep neural network, *LGBM* light gradient boosting machine, *XGB* extreme gradient boosting.

Other performance indices such as accuracy, F1 score, recall, precision, F2 score, specificity, and Matthews correlation coefficient (MCC) for predicting decrease in MAP within 6 h are shown in Table 2. For the outcome of a reduction in MAP ≥ 20 mmHg, the LGBM model achieved the highest accuracy. The support vector machine (SVM) models showed the highest accuracy for predicting a reduction in MAP ≥ 30 mmHg. The XGB models showed the highest F1 score and MCC in predicting a reduction in MAP ≥ 20 mmHg and ≥ 30 mmHg among machine learning models. All of these indices in machine learning models were higher than those in conventional scoring models. When the outcome was defined using other criteria, the machine learning models had the higher AUROC values than the SOFA, APACHE II, and MOSAIC scores: the XGB model when the timeframe was 1 h (Table S4); and nadir MAP was used (Table S5). XGB models showed significantly higher values of AUROCs than logistic regression models for the outcome of a reduction in MAP ≥ 30 mmHg within 1 h, nadir MAP < 65 mmHg, and < 55 mmHg. In addition, XGB models showed higher values of F1 score and MCC than logistic regression models for all outcomes except reduction in MAP ≥ 30 mmHg within 6 h.

**Table 2:** Performance indices including accuracy, F1 score, and Matthews correlation coefficient of models in predicting hypotension within 6 h.

Performance indices	Outcomes	
	MAP Δ20	MAP Δ30
**Accuracy**		
SOFA	0.304	0.203
APACHE II	0.359	0.565
MOSAIC	0.373	0.545
LR	0.769	0.877
SVM	0.745	0.882
DNN	0.749	0.872
LGBM	0.782	0.847
XGB	0.763	0.844
**F1 score**		
SOFA	0.450	0.257
APACHE II	0.461	0.271
MOSAIC	0.453	0.275
LR	0.630	0.588
SVM	0.637	0.570
DNN	0.645	0.587
LGBM	0.637	0.565
XGB	0.660	0.587
**Recall (sensitivity)**		
SOFA	0.966	0.933
APACHE II	0.928	0.548
MOSAIC	0.880	0.587
LR	0.668	0.596
SVM	0.760	0.529
DNN	0.774	0.615
LGBM	0.649	0.673
XGB	0.779	0.750
**Precision**		
SOFA	0.293	0.149
APACHE II	0.306	0.180
MOSAIC	0.305	0.180
LR	0.597	0.579
SVM	0.549	0.618
DNN	0.553	0.561
LGBM	0.625	0.486
XGB	0.572	0.482
**F2 score**		
SOFA	0.662	0.454
APACHE II	0.660	0.389
MOSAIC	0.639	0.404
LR	0.653	0.593
SVM	0.705	0.545
DNN	0.717	0.604
LGBM	0.703	0.599
XGB	0.726	0.675
**Specificity**		
SOFA	0.026	0.077
APACHE II	0.121	0.567
MOSAIC	0.161	0.537
LR	0.811	0.925
SVM	0.738	0.943
DNN	0.738	0.917
LGBM	0.837	0.877
XGB	0.757	0.860
**Matthews correlation coefficient**		
SOFA	–0.021	0.012
APACHE II	0.072	0.082
MOSAIC	0.052	0.088
LR	0.465	0.515
SVM	0.462	0.504
DNN	0.475	0.513
LGBM	0.481	0.484
XGB	0.498	0.514

*MAP* mean arterial pressure, *MAP Δ20* reduction in MAP ≥ 20 mmHg from the initial value, *MAP Δ30* reduction in MAP ≥ 30 mmHg from the initial value, *SOFA* Sequential Organ Failure Assessment, *APACHE* Acute Physiology and Chronic Health Evaluation, *MOSAC* Mortality Scoring system for AKI with CRRT, *SVM* support vector machine, *DNN* deep neural network, *LGBM* light gradient boosting machine, *XGB* extreme gradient boosting.

### Rank of features in machine learning model

**Figure 2:**
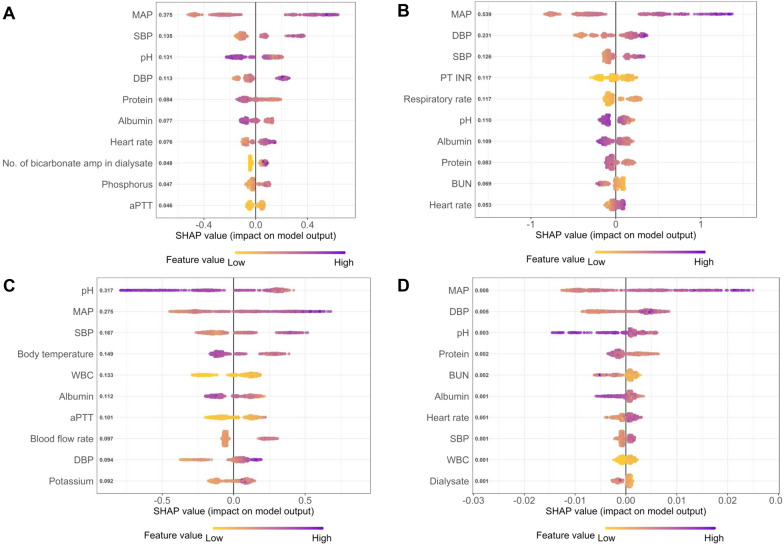
Feature ranking analysis of the light gradient boosting machine model in predicting a reduction in mean arterial pressure (MAP) ≥ 20 mmHg (**A**, **C**) and ≥ 30 mmHg (**B**, **D**) within 6 h (**A**, **B**) and 1 h (**C**, **D**). *MAP* mean arterial pressure, *SBP* systolic blood pressure, *DBP* diastolic blood pressure, *aPTT* activated partial thromboplastin time, *PT-INR* prothrombin time-international normalized ratio, *BUN* blood urea nitrogen, *WBC* white blood cell.

**Figure 3:**
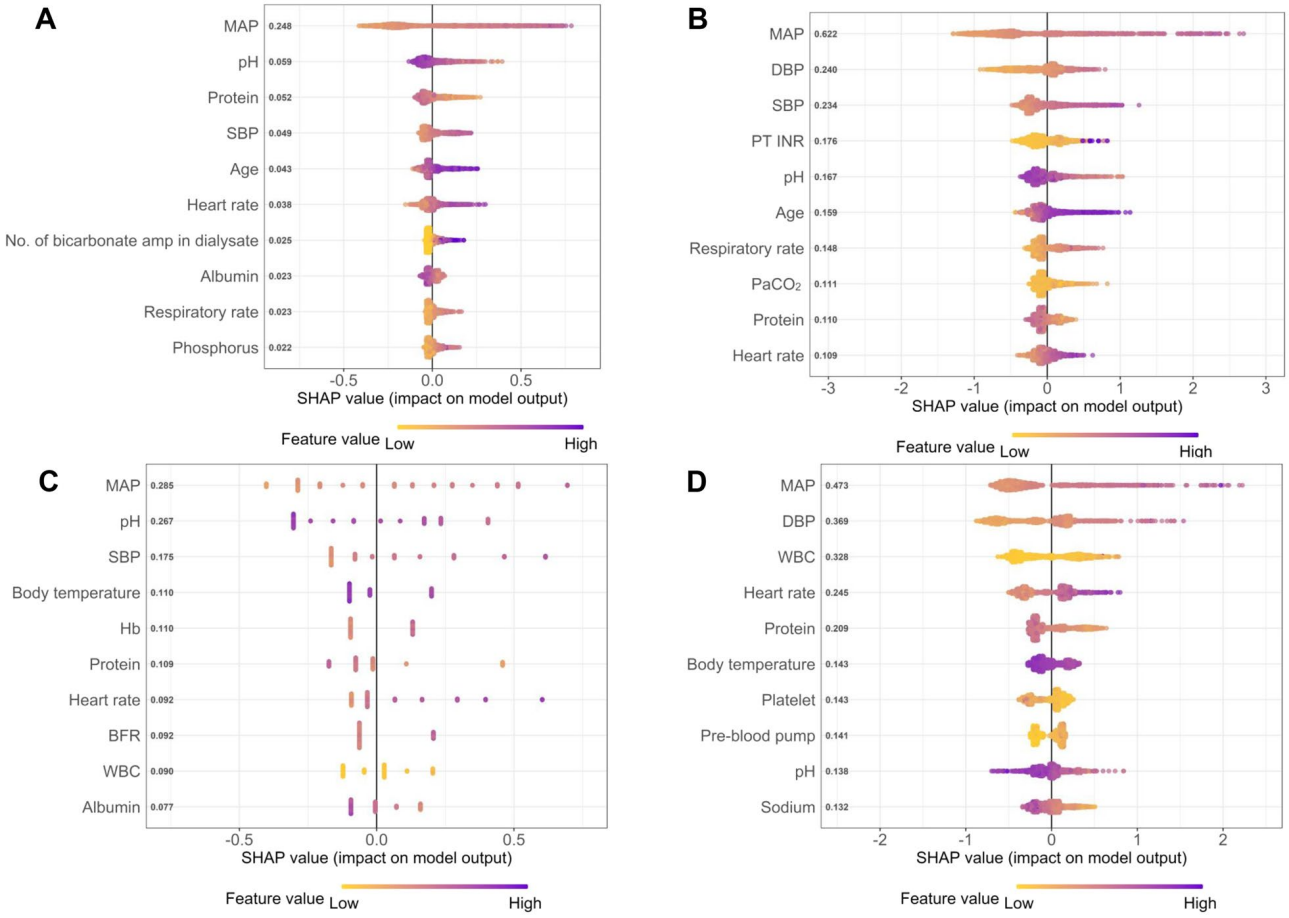
Feature ranking analysis of the extreme gradient boosting machine model in predicting a reduction in mean arterial pressure (MAP) ≥ 20 mmHg (**A**, **C**) and ≥ 30 mmHg (**B**, **D**) within 6 h (**A**, **B**) and 1 h (**C**, **D**). *MAP* mean arterial pressure, *SBP* systolic blood pressure, *DBP* diastolic blood pressure, *PT-INR* prothrombin time-international normalized ratio, *PaCO*_*2*_ arterial partial pressure of carbon dioxide, *Hb* hemoglobin, *BFR* blood flow rate, *WBC* white blood cell.

To estimate the contribution degree of each feature in predicting the risk of hypotension, the feature ranking analysis was performed. The features contributing to the LGBM and XGB models were laboratory findings and vital signs (Figs. 2 and 3). Among laboratory findings, pH was the most important predictor, and serum protein and albumin were the next. Among vital signs, the MAP was the best contributor. In the SVM model, BPs were the most important in predicting MAP drop within 6 h, and some medications were important in predicting MAP drop within 1 h (Fig. S2). In the DNN model, BPs were the most important in the model performance, and other vital signs, pH, and some medications were determined to be important (Fig. S3).

The change in the model performance of XGB and DNN was evaluated by adding each of the top 10 features in order of ranking results of each model (Tables 3 and S6). In the XGB model, the AUROC values increased depending on the features used, whereas the accuracy, F1 score, and, MCC had an increasing trend from 5 to 10 features (Table 3). In the DNN model, increasing performance was shown for the top 30 features used in the model (Table S6). These results indicate that at least 20 or 30 features were needed to precisely predict the hypotension risk in the above machine learning models.

**Table 3:** Performance indices of extreme gradient boosting machine models in predicting hypotension (Defined as a reduction in mean arterial pressure ≥ 20 mmHg from the initial value within 6 h) according to the number of features.

No. of features	AUROC (95% CI)	Accuracy	F1 score	MCC	Brier’s score
5	0.795 (0.759–0.831)	0.691	0.612	0.420	0.159
10	0.813 (0.779–0.848)	0.783	0.642	0.487	0.153
20	0.815 (0.781–0.849)	0.766	0.634	0.467	0.152
30	0.818 (0.785–0.852)	0.756	0.636	0.463	0.154
40	0.820 (0.786–0.853)	0. 787	0.650	0.497	0.155
50	0.823 (0.790–0.856)	0.736	0.642	0.469	0.170
60	0.822 (0.789–0.855)	0.800	0.641	0.505	0.151
70	0.827 (0.794–0.861)	0.792	0.648	0.499	0.148

*AUROC* area under the receiver operating characteristic curve, *CI* confidence interval, *MCC* Matthews correlation coefficient.

### Calibration of models

When Brier’s scores were calculated for calibration, the XGB model had the lowest value for most outcomes, and other models had relatively low values (Table S7). All machine learning models had lower values of Brier’s scores than other conventional scores such as SOFA, APACHE II, and MOSAIC. The XGB models showed the lowest Brier's score among machine learning models predicting outcomes, except predicting the outcome of MAP ≥ 20 mmHg within 6 h. The XGB model had a lower Brier's score than the logistic regression model for the outcomes of reduction in MAP ≥ 20 mmHg and ≥ 30 mmHg within 1 h, and MAP < 65 mmHg and MAP < 55 mmHg within 6 h.

### Models using conventional disease-severity scores as predictors

Table S8 shows the AUROCs of the logistic regression and XGB models using SOFA, APACHE II, and MOSAIC scores as predictors, and their performances seemed to be poor. The XGB models with disease-severity scores in addition to all 92 features also showed lower performance than those with 92 features alone.

### Nested tenfold cross-validation

The AUROC values of machine learning models with the nested tenfold cross-validation were lower than the previous results (Table S9).

## Discussion

Unexpected hypotensive events after starting CRRT are a critical issue because they contribute to worse outcomes, as noted in the above association with high ICU mortality^5,6^. Machine learning models such as XGB, LGBM, and DNN successfully predicted the risk of hypotension and performed better than conventional scoring models such as SOFA, APACHE II, and MOSAIC. The XGB model had the best performance among all models. AUROC was significantly higher in the XGB model than in the logistic regression model only for outcomes of MAP < 65 mmHg, and MAP < 55 mmHg within 6 h, and MAP Δ30 within 1 h. However, the XGB model had higher F1 score and MCC for all outcomes except MAP Δ30 within 6 h than the logistic regression model. These results indicate that precise prediction of CRRT-related hypotension is achievable by machine learning algorithms, especially XGB, although complex and interactive relationships of several features exist.

Based on the ranking analysis, at least 10 features were required to develop machine learning models, and the corresponding 10 features are as shown in the Figs. 3 and S3, including MAP, SBP, DBP, heart rate, pH, serum protein, and prothrombin time-international normalized ratio (PT INR). The value of pH is important to predict hypotension because it is well known that metabolic acidosis frequently causes hypotension^14^. Because the patients with prolonged coagulation time due to sepsis or acute liver failure have a high risk of hypotension, PT INR was important predictor feature^15,16^.

Critically ill patients undergoing CRRT are in a complex clinical situation, which frequently embarrass clinicians in determining the outcomes. Machine learning may overcome the difficulty of considering complex and numerous clinical situations. Several studies have applied machine learning algorithms to critically ill patients and have shown superior performance compared to existing models or scoring systems in predicting outcomes^17^. Our previous study also demonstrated that machine learning had better performance than conventional scoring systems, such as SOFA and APACHE II, in predicting mortality of CRRT patients^10^. The present study expands the utility of machine learning in predicting hypotension as other outcomes of CRRT and provides a clue on advanced management before the occurrence of hypotension.

Excessive ultrafiltration is thought to significantly affect hypotension during CRRT^13^. Other conditions such as reduced cardiac preload resulting from defective vasoconstriction and redistribution of fluids resulting from sepsis or inflammation also contribute to hypotension during CRRT^18,19^. Rapid clearance of plasma solutes by convention method results in osmolar reduction and shifts water from intravascular to interstitial compartments, consequently causing decreased effective arterial blood volume and hypotension^13^. Concurrent cardiac dysfunction can be aggravated by ultrafiltration or blood flow of CRRT, resulting in hypotension^20^. However, precise prediction of CRRT-related hypotension could not be obtained by this theoretical approach alone in real clinical practice. The present feature ranking analysis demonstrated that vital signs at the time of CRRT are the most important contributor to hypotension, which should be assessed before starting CRRT.

Although the results are informative, there are certain limitations to be discussed. Because of a single center design, external validation was not available. The sample size of the cohort was modest. The advantage of machine learning is its high performance, particularly with extremely large sample size. However, there is no specific cutoff on the sample size in machine learning algorithms, and the present sample size of 2349 with ≥ 90 features was greater than the sample size (n = 488) of the previous 258 studies which used machine learning algorithms to analyze ICU data^21^. Because the study analyzed a retrospective cohort, prospective validation is needed. The study identified the most important features with respect to predicting hypotension, but certain degrees of risk, such as the relative risk, could not be obtained. This is a common limitation of machine learning algorithms. Concerns could be raised regarding other issues such as overfitting and the effects of un-identified factors such as response to time-varying vasoactive support and ultrafiltration. The present non-nested cross-validation method could result in a possibility of overfitting.

The SOFA, APACHE II, and MOSAIC scores have been developed to predict mortality but not hypotension after CRRT, which might have low performance.

## Conclusions

The application of machine learning algorithms improves the predictability of hypotension after starting CRRT, and machine learning performs better than conventional scoring models used in critically ill patients. If the machine learning-based prediction models are successfully applied to clinical practice, the overall patient outcomes will improve by proactive management of hypotension. Future studies will explore whether machine learning can predict other outcomes of CRRT and will validate results in an independent cohort.

## Method

### Data source and study subjects

A total of 2,756 adult patients (≥ 18 years old) who started CRRT due to acute kidney injury were retrospectively reviewed at Seoul National University Hospital from June 2010 to February 2020. Patients who had underlying end-stage renal disease (n = 344), stopped CRRT within 1 h after initiation (n = 49), and had no information on comorbidities or laboratory data (n = 14) were excluded. Accordingly, 2349 patients were analyzed in the present study. The patients were randomly divided into a training set (70%) to develop the models and a testing set (30%) to test and calibrate their performance. The study was approved by the institutional review board of the Seoul National University Hospital (no. H-2003-024-1106). All methods have been carried out in accordance with the guidelines, relevant regulations and ethical principles for medical research guided by the Declaration of Helsinki. The requirement of informed consent was waived by the board.

### Study variables and outcomes

Using an electronic medical record system, a total of 92 features were used to develop machine learning models. We used the features before and at the time of starting CRRT during the model development. The features within 24 h prior to starting CRRT were medications, infusion rate of fluids, and laboratory findings. Other features were measured at the time of starting CRRT. Clinical features included age, sex, weight, application of the mechanical ventilator, and comorbidities, such as diabetes mellitus, hypertension, ischemic heart disease, chronic heart failure, stroke, peripheral vascular disease, dementia, chronic kidney disease including diabetic nephropathy, chronic obstructive pulmonary disease, connective tissue disease, peptic ulcer disease, cancer, and arrhythmia including atrial fibrillation, atrioventricular block, ventricular tachycardia, tachycardia-bradycardia syndrome, and total left bundle branch block. Vital signs such as SBP, DBP, MAP, heart rate, respiratory rate, and body temperature were measured at the time of initiating CRRT. The blood pressure values were continuously collected every 1 h or less after starting CRRT. The laboratory data included white blood cell counts, hemoglobin, hematocrit, platelet, total bilirubin, blood urea nitrogen, creatinine, total protein, albumin, pH, sodium, potassium, calcium, phosphate, uric acid, prothrombin time-international normalized ratio, activated partial thromboplastin time, partial pressures of arterial carbon dioxide and oxygen, partial pressure to fractional inspired oxygen, alveolar to arterial oxygen gradient, and the presence of bacteremia. As a setting value, target dose, blood flow rate, amount of dialysate and replacement fluids (pre- and post-dilution), target amount of input and output, the number of bicarbonate ampules mixed in dialysate and replacement fluids, and catheter type were collected. The information on the infused medications or fluids and their infusion rates were obtained, as shown in Table S1. The number of bicarbonate ampules mixed in these fluids were calculated. The Glasgow coma scales were calculated. The SOFA, APACHE II, and MOSAIC scores were measured based on the methods presented in the original studies^22–24^. Hypotension was defined as a reduction in MAP ≥ 20 mmHg from the initial value within 6 h. Additionally, other definitions were used such as a reduction in MAP ≥ 30 mmHg from the initial value, setting the timeframe to within 1 h, or nadir MAP < 55 or 65 mmHg. The ICU mortality, which was defined as all-cause death during the ICU admission, was estimated.

### Statistical analysis and development of machine learning models

Development of machine learning models and statistical analyses were performed using R software (version 4.0.2; The Comprehensive R Archive Network: http://cran.r-project.org). Categorical and continuous features are expressed as proportions and the means ± standard deviation, respectively. The chi-square test was used to compare categorical features (Fisher’s exact test, if not applicable), and the Student’s t test was used to compare continuous features between the training and testing sets. The restricted cubic spline was used to display the odds ratio of ICU mortality according to the change in MAP values during CRRT.

Four machine learning algorithms were used including the SVM, DNN, LGBM, and XGB. We developed machine learning models using a tenfold cross-validation in the training dataset, and the models were evaluated using the test dataset to identify the performance of models. The SVM models used four kernels including linear, polynomial, sigmoid, and radial basis functions. For each kernel, tenfold cross-validation to determine the best set of hyperparameters (cost, gamma, degree, and coefficients) was performed using grid search. The kernels corresponding to the highest AUROC were derived from the final model. In the DNN model (i.e., artificial neural network with multiple layers between the input and output layers), optimal hyperparameters consisting of the size (number of hidden nodes) and decay (parameter for weight decay) with tenfold cross-validation and grid search were determined. When developing the SVM and DNN models, the continuous features were normalized, and categorical features were processed as a one-hot encoding. In the LGBM model, hyperparameters (max_bin, learning rate, and nrounds) were adjusted, and the model with the highest AUROC was selected for comparison. In the XGB model, hyperparameters (eta, gamma, max depth, and nrounds) were adjusted, and the model with the highest AUROC was selected for comparison. For comparing with machine learning models, we have developed logistic regression models predicting outcomes. Machine learning models using SOFA, APACHE II, and MOSAIC scores as predictors were developed and evaluated. To evaluate the suitability of machine learning algorithms to our data and compare among machine learning models, nested tenfold cross-validation was additionally conducted with total study data for predicting reduction in MAP ≥ 20 mmHg and MAP ≥ 30 mmHg from the initial value within 6 h, inner loop with tenfold for hyper-parameter tuning and an outer loop with tenfold for validation of models.

For performance indices, AUROC, F1 score, recall, precision, F2 score, specificity, and MCC were measured in the testing set. The AUROCs were compared between models using the DeLong test. The confidence intervals of AUROCs were estimated using the DeLong method^25,26^. MCC is an informative and truthful score in evaluating binary classification compared to accuracy and F1 score^27^. The MCC values of + 1, 0, and – 1 represent perfect prediction, average random prediction, and inverse prediction, respectively. The threshold was determined when the F1 score was the highest. For calibration, Brier's scores were calculated, with those closer to 0 indicating good calibration. We ranked the importance of features in the SVM with weight vectors, the DNN with weight values, and the LGBM and XGB models with SHapley Additive exPlanations (SHAP)^28–30^. The performance of machine learning models with variable numbers of features in order of ranking were also evaluated. *P* values less than 0.05 were considered significant.

## Supplementary Information

Supplementary Information 1.

Supplementary Information 2.
