# Supplementary material for: Machine learning model to predict hypotension after starting continuous renal replacement therapy
Source: Sci Rep. 2021 Aug 25;11:17169. doi: 10.1038/s41598-021-96727-4 (PMC8387375; doi:10.1038/s41598-021-96727-4)

**Machine learning model to predict hypotension after starting continuous renal replacement therapy**

Min Woo Kang^1^, Seonmi Kim^1^, Yong Chul Kim^1^, Dong Ki Kim^1^, Kook-Hwan Oh^1^, Kwon Wook Joo^1^, Yon Su Kim^1^, and Seung Seok Han^1^

^1^Department of Internal Medicine, Seoul National University College of Medicine, Seoul, Korea

Table S1. Baseline characteristics

| Features | Total  (n = 2,349) | Training set  (n = 1,644) | Testing set  (n = 705) | *P* |
| --- | --- | --- | --- | --- |
| Age (yr) | 64.0 ± 15.0 | 64.0 ± 15.0 | 64.0 ± 15.1 | 0.978 |
| Male (%) | 61.4 | 61.4 | 61.6 | 0.969 |
| Body weight (kg) | 61.7 ± 13.2 | 61.7 ± 13.2 | 61.4 ± 13.2 | 0.606 |
| Systolic blood pressure (mmHg) | 113.7 ± 28.3 | 113.9 ± 28.7 | 113.1 ± 27.5 | 0.526 |
| Diastolic blood pressure (mmHg) | 58.7 ± 15.6 | 56.2 ± 16.5 | 55.5 ± 15.3 | 0.985 |
| Mean arterial pressure (mmHg) | 77.0 ± 17.4 | 72.6 ± 19.3 | 71.6 ± 17.7 | 0.725 |
| Heart rate (/min) | 104.2 ± 25.3 | 104.4 ± 24.9 | 103.9 ± 26.1 | 0.640 |
| Respiratory rate (/min) | 24.0 ± 8.0 | 23.9 ± 7.7 | 24.2 ± 8.7 | 0.404 |
| Body temperature (°C) | 36.4 ± 1.5 | 36.4 ± 1.6 | 36.4 ± 1.3 | 0.927 |
| White blood cells (×10^3^/μl) | 15.1 ± 20.7 | 15.0 ± 21.5 | 15.5 ± 18.6 | 0.567 |
| Hemoglobin (g/dl) | 9.4 ± 2.3 | 9.3 ± 2.2 | 9.4 ± 2.4 | 0.558 |
| Hematocrit (%) | 27.6 ± 7.1 | 27.5 ± 7.0 | 27.8 ± 7.3 | 0.300 |
| Platelet (×10^3^/μl) | 99.4 ± 83.6 | 99.6 ± 85.2 | 98.8 ± 79.6 | 0.836 |
| Calcium (mg/dl) | 7.7 ± 1.4 | 7.7 ± 1.5 | 7.8 ± 1.3 | 0.248 |
| Phosphate (mg/dl) | 6.3 ± 2.5 | 6.2 ± 2.5 | 6.4 ± 2.5 | 0.355 |
| Uric acid (mg/dl) | 7.7 ± 3.4 | 7.8 ± 3.7 | 7.6 ± 3.3 | 0.399 |
| Blood urea nitrogen (mg/dl) | 52.7 ± 30.5 | 52.9 ± 30.6 | 52.2 ± 30.2 | 0.598 |
| Creatinine (mg/dl) | 2.8 ± 2.0 | 2.8 ± 1.9 | 2.8 ± 2.0 | 0.407 |
| Total protein (g/dl) | 4.8 ± 1.1 | 4.8 ± 1.1 | 4.8 ± 1.1 | 0.301 |
| Albumin (g/dl) | 2.7 ± 0.6 | 2.7 ± 0.6 | 2.7 ± 0.6 | 0.787 |
| Total bilirubin (mg/dl) | 4.7 ± 7.4 | 4.7 ± 7.3 | 4.7 ± 7.7 | 0.902 |
| pH | 7.28 ± 0.15 | 7.28 ± 0.16 | 7.28 ± 0.15 | 0.852 |
| Sodium (mmol/l) | 136.4 ± 8.2 | 136.3 ± 8.2 | 136.7 ± 7.9 | 0.236 |
| Potassium (mmol/l) | 4.6 ± 1.1 | 4.6 ± 1.0 | 4.6 ± 1.1 | 0.483 |
| PaCO_2_ (mmHg) | 46.5 ± 17.5 | 46.7 ± 18.1 | 46.1 ± 15.8 | 0.473 |
| PaO_2_ (mmHg) | 112.9 ± 74.6 | 114.5 ± 79.1 | 109.0 ± 62.9 | 0.074 |
| Alveolar–arterial O_2_ gradient | 198.4 ± 244.2 | 199.1 ± 249.7 | 196.9 ± 231.1 | 0.838 |
| PaO_2_/FiO_2_ | 265.4 ± 200.5 | 266.8 ± 206.8 | 262.3 ± 185.0 | 0.605 |
| PT-INR | 2.2 ± 2.3 | 2.2 ± 2.4 | 2.1 ± 2.0 | 0.460 |
| aPTT (sec) | 60.5 ± 59.5 | 61.4 ± 61.6 | 58.4 ± 54.4 | 0.244 |
| Target clearance (ml/min) | 40.7 ± 13.1 | 40.6 ± 13.3 | 40.8 ± 12.7 | 0.714 |
| Blood flow rate (ml/min) | 110.6 ± 24.6 | 110.1 ± 24.3 | 111.8 ± 25.4 | 0.115 |
| Dialysate setting (ml/hr) | 1281.7 ± 503.0 | 1278.0 ± 504.2 | 1290.1 ± 500.5 | 0.594 |
| Pre-blood pump setting (ml/hr) | 741.4 ± 660.5 | 750.6 ± 663.1 | 719.9 ± 654.6 | 0.300 |
| Replacement setting (ml/hr) | 988.9 ± 687.5 | 970.9 ± 696.2 | 1030.9 ± 665.5 | 0.052 |
| Target input/output (ml/d) | –337.0 ± 913.7 | –335.0 ± 947.8 | –341.7 ± 839.3 | 0.869 |
| No. of HCO_3_^–^ amp in dialysate | 1.4 ± 2.1 | 1.4 ± 2.1 | 1.4 ± 2.1 | 0.952 |
| No. of HCO_3_^–^ amp in pre-dilution replacement | 1.0 ± 1.9 | 1. ± 1.9 | 1.0 ± 1.9 | 0.723 |
| No. of HCO_3_^–^ amp in post-dilution replacement | 0.6 ± 1.6 | 0.6 ± 1.6 | 0.6 ± 1.6 | 0.905 |
| Dobutamine infusion rate (μg/kg/min) | 1.2 ± 4.2 | 1.2 ± 4.1 | 1.2 ± 4.4 | 0.823 |
| Dopamine infusion rate (μg/kg/min) | 3.0 ± 8.1 | 3.0 ± 8.0 | 3.0 ± 8.2 | 0.985 |
| Norepinephrine infusion rate (μg/min) | 10.9 ± 20.0 | 10.7 ± 20.2 | 11.6 ± 19.6 | 0.332 |
| Vasopressin infusion rate (unit/min) | 0.005 ± 0.024 | 0.005 ± 0.024 | 0.005 ± 0.023 | 0.794 |
| Epinephrine infusion rate (μg/kg/min) | 0.23 ± 1.83 | 0.24 ± 2.00 | 0.19 ± 1.37 | 0.561 |
| Nitroglycerin infusion rate (μg/min) | 2.40 ± 11.51 | 2.22 ± 10.86 | 2.81 ± 12.89 | 0.256 |
| Milrinone infusion rate (μg/kg/min) | 0.009 ± 0.65 | 0.009 ± 0.07 | 0.009 ± 0.07 | 0.939 |
| Diltiazem infusion rate (mg/hr) | 0.13 ± 1.15 | 0.12 ± 1.11 | 0.15 ± 1.25 | 0.590 |
| Esmolol infusion rate (μg/kg/min) | 0.40 ± 5.09 | 0.47 ± 5.50 | 0.23 ± 3.97 | 0.295 |
| Nicorandil infusion rate (mg/hr) | 0.01 ± 0.16 | 0.02 ± 0.17 | 0.01 ± 0.12 | 0.302 |
| Nicardipine infusion rate (mg/hr) | 0.04 ± 0.47 | 0.03 ± 0.42 | 0.05 ± 0.59 | 0.518 |
| 0.9% saline infusion rate (ml/hr) | 13.7 ± 33.1 | 13.4 ± 32.2 | 14.3 ± 35.0 | 0.554 |
| Hartmann solution infusion rate (ml/hr) | 3.6 ± 18.1 | 3.4 ± 17.3 | 4.0 ± 19.7 | 0.462 |
| Plasma solution A infusion rate (ml/hr) | 2.9 ± 15.8 | 3.3 ± 16.8 | 2.0 ± 13.1 | 0.064 |
| Half saline infusion rate (ml/hr) | 1.8 ± 12.4 | 1.7 ± 11.9 | 2.1 ± 13.4 | 0.519 |
| 3% saline infusion rate (ml/hr) | 0.2 ± 2.2 | 0.2 ± 2.5 | 0.1 ± 1.3 | 0.271 |
| Hydroxyethyl starch infusion rate (ml/hr) | 0.2 ± 3.7 | 0.3 ± 4.4 | 0.1 ± 1.1 | 0.117 |
| D5W infusion rate (ml/hr) | 16.7 ± 37.1 | 17.0 ± 38.7 | 15.8 ± 32.9 | 0.464 |
| D10W infusion rate (ml/hr) | 7.3 ± 22.0 | 7.3 ± 21.7 | 7.3 ± 22.7 | 0.982 |
| D50W infusion rate (ml/hr) | 0.4 ± 3.8 | 0.4 ± 3.9 | 0.5 ± 3.8 | 0.306 |
| Dextrose saline infusion rate (ml/hr) | 1.7 ± 11.4 | 1.7 ± 11.3 | 1.7 ± 11.5 | 0.860 |
| NK2 infusion rate (ml/hr) | 1.8 ± 11.2 | 1.6 ± 11.3 | 2.1 ± 11.2 | 0.310 |
| Hartmann-D solution infusion rate (ml/hr) | 0.5 ± 5.8 | 0.4 ± 5.2 | 0.7 ± 7.0 | 0.332 |
| No. of HCO_3_^–^ amp in main fluids | 1.4 ± 2.7 | 1.4 ± 2.7 | 1.4 ± 2.7 | 0.879 |
| Glasgow coma scale | 8.0 ± 4.5 | 8.0 ± 4.5 | 8.0 ± 4.5 | 0.801 |
| Bacteremia (%) | 22.6 | 21.8 | 24.5 | 0.142 |
| Diabetes mellitus (%) | 28.6 | 29.1 | 27.4 | 0.387 |
| Hypertension (%) | 26.4 | 26.1 | 27.2 | 0.566 |
| Myocardial infarction (%) | 9.3 | 9.2 | 9.4 | 0.929 |
| Chronic heart failure (%) | 16.1 | 16.1 | 16.2 | 0.946 |
| Stroke (%) | 13.3 | 13.1 | 13.8 | 0.685 |
| Peripheral vascular disease (%) | 5.4 | 5.3 | 5.7 | 0.708 |
| Dementia (%) | 5.1 | 5.0 | 5.2 | 0.840 |
| Diabetic nephropathy (%) | 3.0 | 3.1 | 2.7 | 0.595 |
| Chronic obstructive pulmonary disease (%) | 4.9 | 5.5 | 3.7 | 0.067 |
| Connective tissue disease (%) | 1.2 | 1.3 | 1.1 | 0.774 |
| Peptic ulcer disease (%) | 3.8 | 3.8 | 3.8 | 0.998 |
| Cancer (%) | 38.0 | 37.2 | 39.9 | 0.229 |
| Ischemic heart disease (%) | 11.2 | 11.8 | 9.8 | 0.156 |
| Chronic kidney disease (%) | 22.5 | 21.5 | 24.8 | 0.080 |
| Atrial fibrillation (%) | 12.4 | 12.5 | 12.3 | 0.931 |
| Atrioventricular block (%) | 1.4 | 1.6 | 1.1 | 0.406 |
| Ventricular tachycardia (%) | 1.8 | 1.8 | 1.8 | 0.975 |
| Tachycardia-bradycardia syndrome (%) | 0.3 | 0.2 | 0.4 | 0.285 |
| Complete left bundle branch block (%) | 0.2 | 0.2 | 0.1 | 0.625 |
| Ventilator apply (%) | 78.2 | 78.2 | 78.4 | 0.882 |
| 5% albumin infusion (%) | 0.9 | 1.0 | 0.6 | 0.327 |
| 20% albumin infusion (%) | 0.6 | 0.7 | 0.4 | 0.482 |
| Catheter (%) |  |  |  |  |
| Internal jugular | 34.3 | 34.1 | 34.6 | 0.820 |
| Femoral | 51.9 | 51.7 | 52.3 | 0.777 |
| Subclavian | 10.6 | 11.1 | 9.5 | 0.241 |
| Subcutaneously-tunneled | 2.7 | 2.5 | 3.3 | 0.294 |
| ECMO | 0.4 | 0.5 | 0.3 | 0.489 |
| SOFA score | 11.9 ± 3.7 | 11.9 ± 3.7 | 12.0 ± 3.7 | 0.837 |
| APACHE II score | 26.4 ± 7.8 | 26.3 ± 7.7 | 26.5 ± 7.9 | 0.641 |
| MOSAIC score | 21.2 ± 10.8 | 21.1 ± 10.8 | 21.3 ± 10.8 | 0.769 |

Abbreviations: PaCO_2_, arterial partial pressure of carbon dioxide; PaO_2_, arterial partial pressure of oxygen; FiO_2_, fraction of inspired oxygen; D5W, 5% dextrose water; D10W, 10% dextrose water; D50W, 50% dextrose water; NK2, 5% dextrose water with 77 mmol/l of sodium and 20 mmol/l of potassium; ECMO, extracorporeal membrane oxygenation; APACHE, Acute Physiology and Chronic Health Evaluation; SOFA, Sequential Organ Failure Assessment; MOSAC, Mortality Scoring system for AKI with CRRT.

Table S2. Area under the receiver operating characteristic curves of the models predicting hypotension within 1 hour

|  | Outcomes | | | | | | | | | |
| --- | --- | --- | --- | --- | --- | --- | --- | --- | --- | --- |
| Models | MAP Δ20 | *P** | *P*^†^ | *P*^‡^ | *P*^§^ | MAP Δ30 | *P** | *P*^†^ | *P*^‡^ | *P*^§^ |
| SOFA | 0.522 (0.456–0.587) |  |  |  |  | 0.533 (0.438–0.628) |  |  |  |  |
| APACHE II | 0.533 (0.466–0.600) |  |  |  |  | 0.612 (0.524–0.700) |  |  |  |  |
| MOSAIC | 0.555 (0.488–0.623) |  |  |  |  | 0.578 (0.487–0.670) |  |  |  |  |
| LR | 0.704 (0.638-0.769) |  |  |  |  | 0.738 (0.639-0.837) |  |  |  |  |
| SVM | 0.696 (0.629–0.764) | <0.001 | <0.001 | <0.001 | 0.755 | 0.729 (0.629-0.828) | 0.002 | <0.001 | <0.001 | 0.051 |
| DNN | 0.743 (0.686–0.801) | <0.001 | <0.001 | <0.001 | 0.376 | 0.774 (0.689–0.860) | 0.010 | <0.001 | 0.002 | 0.498 |
| LGBM | 0.730 (0.674–0.786) | <0.001 | <0.001 | <0.001 | 0.374 | 0.812 (0.742–0.881) | 0.002 | <0.001 | <0.001 | 0.060 |
| XGB | 0.781 (0.729–0.833) | <0.001 | <0.001 | <0.001 | 0.071 | 0.832 (0.760–0.905) | <0.001 | <0.001 | <0.001 | 0.013 |

*Compared with the APACHE II model.

†Compared with the SOFA model.

^‡^Compared with the MOSAIC model.

§ Compared with the LR model

Abbreviations: MAP, mean arterial pressure; MAP Δ20, reduction in MAP ≥20 mmHg from the initial value; MAP Δ30, reduction in MAP ≥30 mmHg from the initial value; SOFA, Sequential Organ Failure Assessment; APACHE, Acute Physiology and Chronic Health Evaluation; MOSAC, Mortality Scoring system for AKI with CRRT; LR, Logistic regression; SVM, support vector machine; DNN, deep neural network; LGBM, light gradient boosting machine; XGB, extreme gradient boosting.

Table S3. Area under the receiver operating characteristic curves of the models predicting hypotension within 6 hours

|  | Outcomes | | | | | | | | | |
| --- | --- | --- | --- | --- | --- | --- | --- | --- | --- | --- |
| Models | Nadir MAP <65 mmHg | *P** | *P*^†^ | *P*^‡^ | *P*^§^ | Nadir MAP <55 mmHg | *P** | *P*^†^ | *P*^‡^ | *P*^§^ |
| SOFA | 0.536 (0.492–0.580) |  |  |  |  | 0.567 (0.519–0.616) |  |  |  |  |
| APACHE II | 0.507 (0.434–0.551) |  |  |  |  | 0.592 (0.542–0.641) |  |  |  |  |
| MOSAIC | 0.552 (0.509–0.595) |  |  |  |  | 0.621 (0.575–0.667) |  |  |  |  |
| LR | 0.604 (0.561-0.647) |  |  |  |  | 0.661 (0.616-0.707) |  |  |  |  |
| SVM | 0.606 (0.563–0.649) | <0.001 | 0.018 | 0.044 | 0.945 | 0.635 (0.588–0.683) | 0.150 | 0.039 | 0.610 | 0.103 |
| DNN | 0.607 (0.565–0.651) | 0.001 | 0.022 | 0.075 | 0.909 | 0.670 (0.625–0.716) | 0.022 | 0.003 | 0.135 | 0.786 |
| LGBM | 0.802 (0.770–0.833) | <0.001 | <0.001 | <0.001 | <0.001 | 0.768 (0.730–0.807) | <0.001 | <0.001 | <0.001 | <0.001 |
| XGB | 0.821 (0.791–0.851) | <0.001 | <0.001 | <0.001 | <0.001 | 0.776 (0.737–0.815) | <0.001 | <0.001 | <0.001 | <0.001 |

*Compared with the APACHE II model.

†Compared with the SOFA model.

^‡^Compared with the MOSAIC model.

§ Compared with the LR model

Abbreviations: SOFA, Sequential Organ Failure Assessment; APACHE, Acute Physiology and Chronic Health Evaluation; MOSAC, Mortality Scoring system for AKI with CRRT; LR, Logistic regression; SVM, support vector machine; DNN, deep neural network; LGBM, light gradient boosting machine; XGB, extreme gradient boosting.

Table S4. Parameters of the model performance in predicting hypotension within 1 hour

|  | Outcomes | |
| --- | --- | --- |
| Performance indices | MAP Δ20 | MAP Δ30 |
| Accuracy |  |  |
| SOFA | 0.321 | 0.538 |
| APACHE II | 0.204 | 0.743 |
| MOSAIC | 0.535 | 0.792 |
| LR | 0.810 | 0.896 |
| SVM | 0.762 | 0.821 |
| DNN | 0.830 | 0.933 |
| LGBM | 0.766 | 0.916 |
| XGB | 0.775 | 0.929 |
| F1 score |  |  |
| SOFA | 0.206 | 0.109 |
| APACHE II | 0.209 | 0.126 |
| MOSAIC | 0.215 | 0.098 |
| LR | 0.350 | 0.305 |
| SVM | 0.339 | 0.250 |
| DNN | 0.375 | 0.356 |
| LGBM | 0.337 | 0.306 |
| XGB | 0.418 | 0.359 |
| Recall (sensitivity) |  |  |
| SOFA | 0.785 | 0.541 |
| APACHE II | 0.937 | 0.351 |
| MOSAIC | 0.570 | 0.216 |
| LR | 0.456 | 0.432 |
| SVM | 0.544 | 0.568 |
| DNN | 0.456 | 0.351 |
| LGBM | 0.532 | 0.351 |
| XGB | 0.722 | 0.378 |
| Precision |  |  |
| SOFA | 0.118 | 0.061 |
| APACHE II | 0.118 | 0.076 |
| MOSAIC | 0.133 | 0.063 |
| LR | 0.283 | 0.235 |
| SVM | 0.246 | 0.160 |
| DNN | 0.319 | 0.361 |
| LGBM | 0.247 | 0.271 |
| XGB | 0.294 | 0.341 |
| F2 score |  |  |
| SOFA | 0.369 | 0.210 |
| APACHE II | 0.391 | 0.204 |
| MOSAIC | 0.344 | 0.146 |
| LR | 0.406 | 0.370 |
| SVM | 0.438 | 0.376 |
| DNN | 0.420 | 0.353 |
| LGBM | 0.451 | 0.393 |
| XGB | 0.559 | 0.370 |
| Specificity |  |  |
| SOFA | 0.262 | 0.537 |
| APACHE II | 0.112 | 0.765 |
| MOSAIC | 0.530 | 0.823 |
| LR | 0.855 | 0.922 |
| SVM | 0.789 | 0.835 |
| DNN | 0.877 | 0.966 |
| LGBM | 0.800 | 0.948 |
| XGB | 0.781 | 0.960 |
| Matthews correlation coefficient |  |  |
| SOFA | 0.034 | 0.035 |
| APACHE II | 0.050 | 0.061 |
| MOSAIC | 0.063 | 0.023 |
| LR | 0.255 | 0.268 |
| SVM | 0.243 | 0.231 |
| DNN | 0.286 | 0.321 |
| LGBM | 0.241 | 0.265 |
| XGB | 0.355 | 0.322 |

Abbreviations: MAP, mean arterial pressure; MAP Δ20, reduction in MAP ≥20 mmHg from the initial value; MAP Δ30, reduction in MAP ≥30 mmHg from the initial value; SOFA, Sequential Organ Failure Assessment; APACHE, Acute Physiology and Chronic Health Evaluation; MOSAC, Mortality Scoring system for AKI with CRRT; LR, Logistic regression; SVM, support vector machine; DNN, deep neural network; LGBM, light gradient boosting machine; XGB, extreme gradient boosting.

Table S5. Parameters of the model performance in predicting hypotension within 6 hours

|  | Outcomes | |
| --- | --- | --- |
| Performance indices | Nadir MAP <65 mmHg | Nadir MAP <55 mmHg |
| Accuracy |  |  |
| SOFA | 0.383 | 0.365 |
| APACHE II | 0.404 | 0.401 |
| MOSAIC | 0.482 | 0.589 |
| LR | 0.441 | 0.562 |
| SVM | 0.448 | 0.556 |
| DNN | 0.454 | 0.627 |
| LGBM | 0.731 | 0.702 |
| XGB | 0.745 | 0.706 |
| F1 score |  |  |
| SOFA | 0.552 | 0.391 |
| APACHE II | 0.557 | 0.401 |
| MOSAIC | 0.561 | 0.427 |
| LR | 0.563 | 0.461 |
| SVM | 0.560 | 0.432 |
| DNN | 0.561 | 0.451 |
| LGBM | 0.684 | 0.535 |
| XGB | 0.722 | 0.541 |
| Recall (sensitivity) |  |  |
| SOFA | 1.000 | 0.862 |
| APACHE II | 0.985 | 0.844 |
| MOSAIC | 0.869 | 0.647 |
| LR | 0.948 | 0.790 |
| SVM | 0.922 | 0.713 |
| DNN | 0.918 | 0.647 |
| LGBM | 0.769 | 0.725 |
| XGB | 0.873 | 0.731 |
| Precision |  |  |
| SOFA | 0.381 | 0.253 |
| APACHE II | 0.388 | 0.263 |
| MOSAIC | 0.414 | 0.319 |
| LR | 0.401 | 0.325 |
| SVM | 0.402 | 0.310 |
| DNN | 0.404 | 0.346 |
| LGBM | 0.617 | 0.425 |
| XGB | 0.616 | 0.430 |
| F2 score |  |  |
| SOFA | 0.755 | 0.582 |
| APACHE II | 0.753 | 0.585 |
| MOSAIC | 0.713 | 0.536 |
| LR | 0.744 | 0.615 |
| SVM | 0.732 | 0.566 |
| DNN | 0.732 | 0.551 |
| LGBM | 0.793 | 0.588 |
| XGB | 0.806 | 0.641 |
| Specificity |  |  |
| SOFA | 0.005 | 0.210 |
| APACHE II | 0.048 | 0.264 |
| MOSAIC | 0.245 | 0.571 |
| LR | 0.130 | 0.491 |
| SVM | 0.158 | 0.507 |
| DNN | 0.169 | 0.621 |
| LGBM | 0.707 | 0.695 |
| XGB | 0.666 | 0.699 |
| Matthews correlation coefficient |  |  |
| SOFA | 0.042 | 0.078 |
| APACHE II | 0.087 | 0.108 |
| MOSAIC | 0.138 | 0.185 |
| LR | 0.126 | 0.242 |
| SVM | 0.116 | 0.188 |
| DNN | 0.123 | 0.229 |
| LGBM | 0.463 | 0.364 |
| XGB | 0.525 | 0.372 |

Abbreviations: MAP, mean arterial pressure; SOFA, Sequential Organ Failure Assessment; APACHE, Acute Physiology and Chronic Health Evaluation; MOSAC, Mortality Scoring system for AKI with CRRT; LR, Logistic regression; SVM, support vector machine; DNN, deep neural network; LGBM, light gradient boosting machine; XGB, extreme gradient boosting.

Table S6. Performance indices of deep neural network models in predicting hypotension* according to the number of features

| No. of features | AUROC (95% CI) | Accuracy | F1 score | MCC | Brier’s score |
| --- | --- | --- | --- | --- | --- |
| 5 | 0.799 (0.763–0.835) | 0.765 | 0.623 | 0.454 | 0.192 |
| 10 | 0.823 (0.789–0.856) | 0.762 | 0.647 | 0.480 | 0.213 |
| 20 | 0.803 (0.767–0.839) | 0.770 | 0.627 | 0.462 | 0.233 |
| 30 | 0.824 (0.790–0.858) | 0.782 | 0.652 | 0.495 | 0.188 |
| 40 | 0.822 (0.788–0.856) | 0.784 | 0.653 | 0.498 | 0.188 |
| 50 | 0.816 (0.781–0.850) | 0.750 | 0.630 | 0.454 | 0.206 |
| 60 | 0.824 (0.791–0.858) | 0.796 | 0.645 | 0.502 | 0.178 |
| 70 | 0.817 (0.783–0.851) | 0.775 | 0.640 | 0.478 | 0.202 |

*Defined as a reduction in mean arterial pressure ≥20 mmHg from the initial value within 6 hours.

Abbreviations: AUROC, area under the receiver operating characteristic curve; CI, confidence intervals; MCC, Matthews correlation coefficient; XGB, extreme gradient boosting.

Table S7. Brier’s score of models

|  | Outcomes | | | | | |
| --- | --- | --- | --- | --- | --- | --- |
| Models | MAP Δ20  within 6 hours | MAP Δ30  within 6 hours | MAP Δ20  within 1 hour | MAP Δ30  within 1 hour | Nadir MAP <65 within 6 hours | Nadir MAP <55 within 6 hours |
| SOFA | 0.208 | 0.126 | 0.099 | 0.049 | 0.235 | 0.179 |
| APACHE II | 0.207 | 0.124 | 0.099 | 0.049 | 0.236 | 0.177 |
| MOSAIC | 0.206 | 0.125 | 0.099 | 0.050 | 0.234 | 0.177 |
| LR | 0.153 | 0.089 | 0.094 | 0.049 | 0.231 | 0.179 |
| SVM | 0.190 | 0.251 | 0.096 | 0.048 | 0.227 | 0.181 |
| DNN | 0.177 | 0.248 | 0.102 | 0.171 | 0.251 | 0.170 |
| LGBM | 0.186 | 0.150 | 0.171 | 0.050 | 0.183 | 0.192 |
| XGB | 0.179 | 0.091 | 0.089 | 0.045 | 0.175 | 0.149 |

Abbreviations: MAP, mean arterial pressure; MAP Δ20, reduction in MAP ≥20 mmHg from the initial value; MAP Δ30, reduction in MAP ≥30 mmHg from the initial value; LR, Logistic regression; SVM, support vector machine; DNN, deep neural network; LGBM, light gradient boosting machine; XGB, extreme gradient boosting.

Table S8. AUROC of models using SOFA, APACHE II and MOSAIC scores

|  | Outcomes | | | | | |
| --- | --- | --- | --- | --- | --- | --- |
| Models | MAP Δ20  within 6 hours | MAP Δ30  within 6 hours | MAP Δ20  within  1 hour | MAP Δ30  within  1 hour | Nadir MAP <65 within 6 hours | Nadir MAP <55 within 6 hours |
| LR | 0.577  (0.529-0.624) | 0.627  (0.568-0.686) | 0.552  (0.484-0.621) | 0.598  (0.502-0.695) | 0.551  (0.508-0.594) | 0.619  (0.572-0.666) |
| XGB* | 0.576  (0.530-0.622) | 0.590  (0.533-0.647) | 0.541  (0.474-0.609) | 0.553  (0.461-0.645) | 0.572  (0.529-0.616) | 0.624  (0.577-0.671) |
| XGB^†^ | 0.807  (0.772-0.841) | 0.856  (0.816-0.896) | 0.715  (0.655-0.774) | 0.816  (0.742-0.890) | 0.821  (0.791-0.851) | 0.774  (0.735-0.813) |

* Using only SOFA, APACHE II and MOSAIC scores as predictive variables.

†Using all variables and added SOFA, APACHE II and MOSAIC scores as predictive variables.

Abbreviations: AUROC, area under the receiver operating characteristic curve; SOFA, Sequential Organ Failure Assessment; APACHE, Acute Physiology and Chronic Health Evaluation; MOSAC, Mortality Scoring system for AKI with CRRT; LR, Logistic regression; XGB, extreme gradient boosting.

|  | Outcomes | |
| --- | --- | --- |
| Models | MAP Δ20 within 6 hours | MAP Δ30 within 6 hours |
| SVM | 0.774 | 0.814 |
| DNN | 0.770 | 0.818 |
| LGBM | 0.771 | 0.817 |
| XGB | 0.775 | 0.817 |

Table S9. Mean area under the receiver operating characteristic curves of models of outer loop in nested 10-fold cross-validation

Abbreviations: MAP, mean arterial pressure; MAP Δ20, reduction in MAP ≥20 mmHg from the initial value; MAP Δ30, reduction in MAP ≥30 mmHg from the initial value; SVM, support vector machine; DNN, deep neural network; LGBM, light gradient boosting machine; XGB, extreme gradient boosting.

Figure S1. Nonlinear relationship between the odds ratio (OR) of intensive care unit (ICU) mortality and the reduction in mean arterial pressure (MAP) within 6 hours (A) and 1 hour (B). Gray area indicates 95% confidence intervals.

(A)


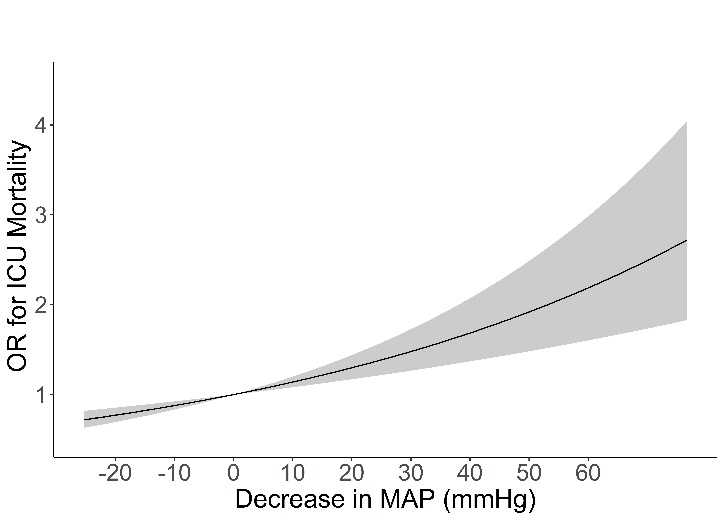


(B)


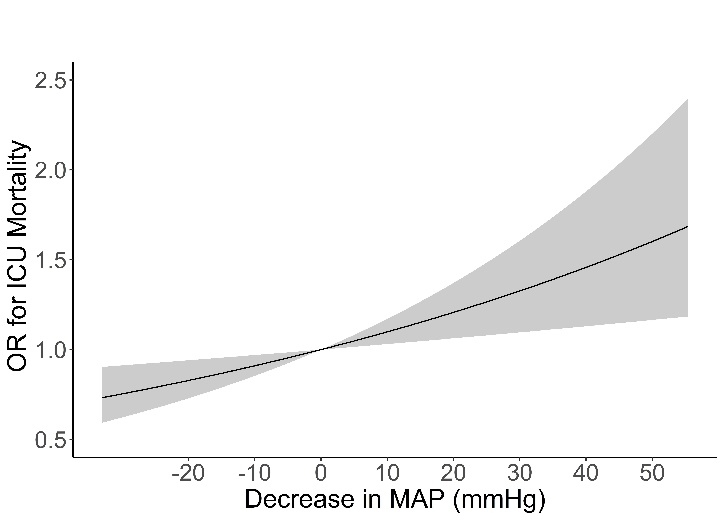


Figure S2. Feature rankings for the support vector machine model in predicting a reduction in mean arterial pressure ≥20 mmHg (A, C) and ≥30 mmHg (B, D) within 6 hours (A, B) and 1 hour (C, D). SBP, systolic blood pressure; MAP, mean arterial pressure; DBP, diastolic blood pressure; BUN, blood urea nitrogen; PBP, Pre-blood pump.

(A)


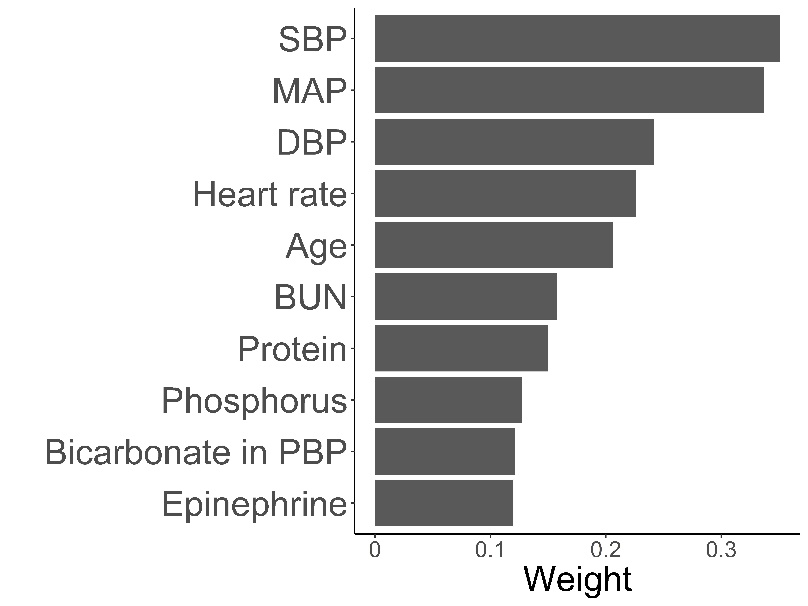


(B)


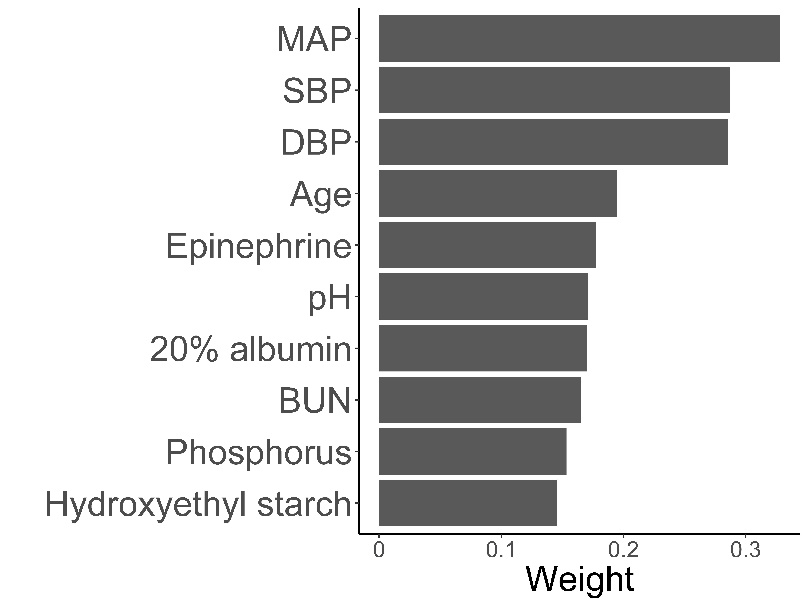


(C)


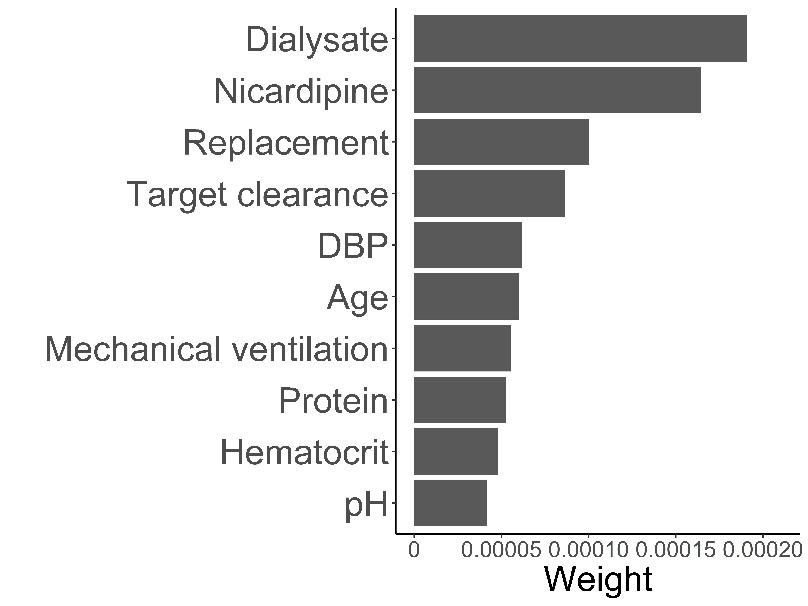


(D)


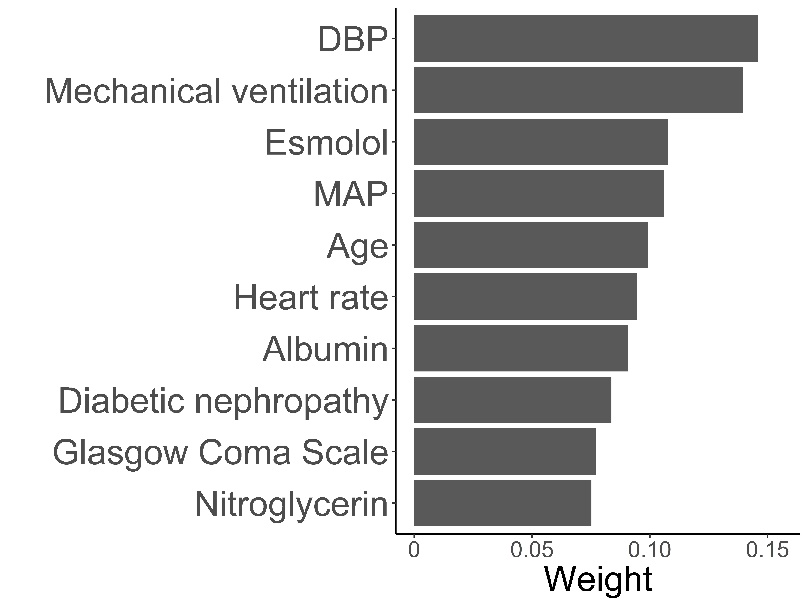


Figure S3. Feature rankings for the deep neural network model in predicting a reduction in mean arterial pressure ≥20 mmHg (A, C) and ≥30 mmHg (B, D) within 6 hours (A, B) and 1 hour (C, D). SBP, systolic blood pressure; MAP, mean arterial pressure; DBP, diastolic blood pressure; BUN, blood urea nitrogen; I/O, input and output; PaO_2_, arterial partial pressure of oxygen; FiO_2_, fraction of inspired oxygen.

(A)


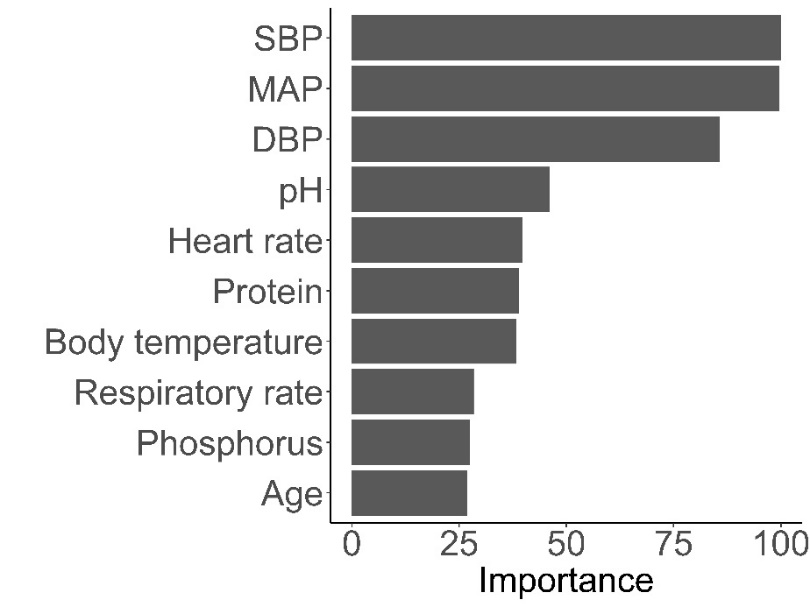


(B)


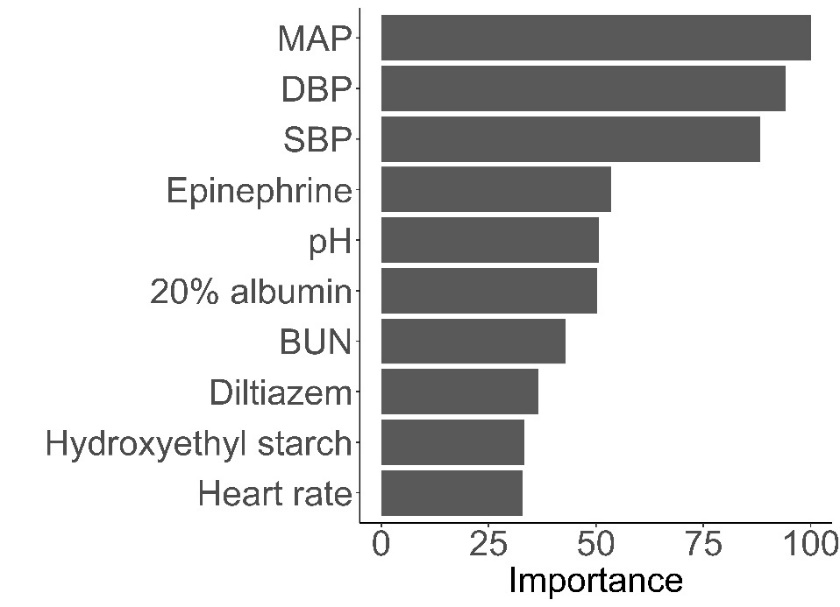


(C)


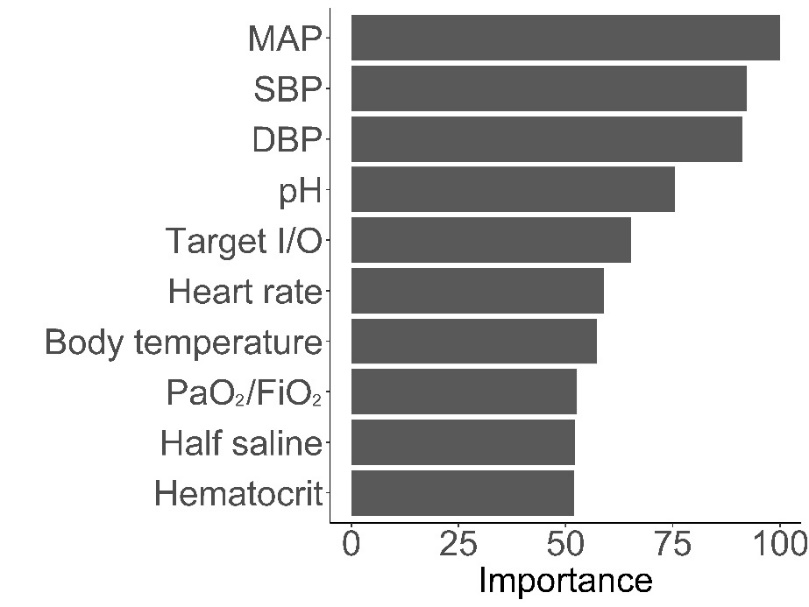


(D)


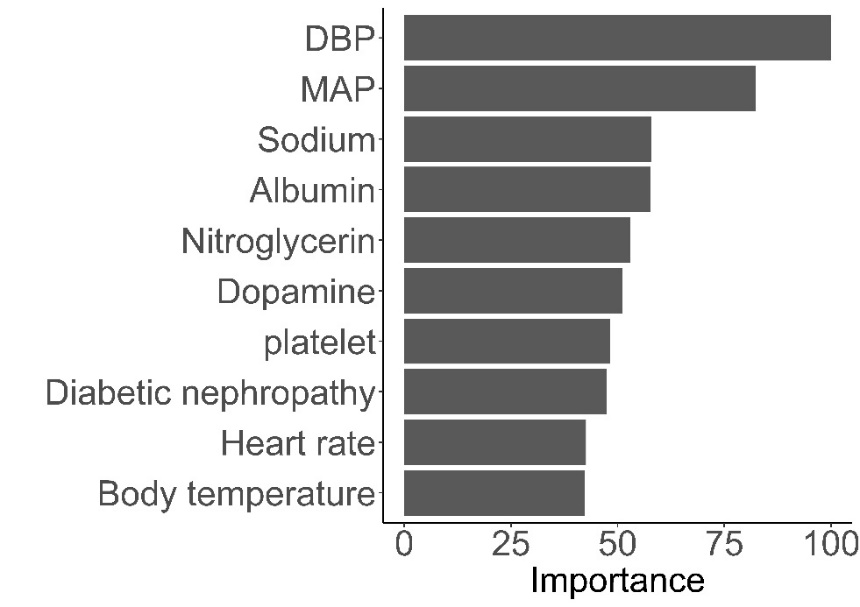

Supplement: Supplementary file 1 — Supplementary Information 1. [file 41598_2021_96727_MOESM1_ESM.docx]
