# Supplementary material for: Machine learning model to predict hypotension after starting continuous renal replacement therapy
Source: Sci Rep. 2021 Aug 25;11:17169. doi: 10.1038/s41598-021-96727-4 (PMC8387375; doi:10.1038/s41598-021-96727-4)
Supplement: Supplementary file 2 — Supplementary Information 2. [file 41598_2021_96727_MOESM2_ESM.docx]

|  | Outcomes | | | | | |
| --- | --- | --- | --- | --- | --- | --- |
| Models | MAP Δ20  within 6 hours | MAP Δ30  within 6 hours | MAP Δ20  within 1 hour | MAP Δ30  within 1 hour | Nadir MAP <65 within 6 hours | Nadir MAP <55 within 6 hours |
| SVM |  |  |  |  |  |  |
| kernel | linear | linear | linear | linear | linear | linear |
| gamma | 0.001 | 0.001 | 0.001 | 0.001 | 0.001 | 0.001 |
| coef | 0.00001 | 0.00001 | 0.00001 | 0.00001 | 0.00001 | 0.00001 |
| DNN |  |  |  |  |  |  |
| size | 1 | 1 | 1 | 1 | 3 | 1 |
| decay | 1.0 | 0.2 | 0.3 | 0.1 | 0.2 | 1.3 |
| LGBM |  |  |  |  |  |  |
| learning rate | 0.005 | 0.005 | 0.005 | 0.0001 | 0.005 | 0.005 |
| max bin | 10000 | 10000 | 10000 | 10000 | 10000 | 10000 |
| nrounds | 829 | 913 | 1051 | 30 | 630 | 664 |
| XGB |  |  |  |  |  |  |
| eta | 0.01 | 0.05 | 0.1 | 0.1 | 0.01 | 0.1 |
| gamma | 0.0000001 | 0.1 | 0.1 | 0.00001 | 0.000000001 | 0.0001 |
| max depth | 10 | 9 | 1 | 2 | 6 | 2 |
| nrounds | 75 | 100 | 100 | 100 | 75 | 100 |

Table 1. Hyperparameters of machine learning models

Abbreviations: MAP Δ20, reduction in MAP ≥20 mmHg from the initial value; MAP Δ30, reduction in MAP ≥30 mmHg from the initial value; SVM, support vector machine; DNN, deep neural network; LGBM, light gradient boosting machine; XGB, extreme gradient boosting.
